# Trends in cutaneous squamous cell carcinoma on the lip incidence and mortality in the United States, 2000–2019

**DOI:** 10.3389/fonc.2023.1111907

**Published:** 2023-04-17

**Authors:** Jin Zhang, Quyang Yang, Jinyan Wu, Ruoyue Yuan, Xiansheng Zhao, Yue Li, Xiujun Cheng, Baojin Wu, Ningwen Zhu

**Affiliations:** ^1^ Department of Dermatology, Shanghai Institute of Dermatology, Huashan Hospital, Fudan University, Shanghai, China; ^2^ Huashan Hospital, Fudan University, Shanghai, China

**Keywords:** cutaneous squamous cell carcinoma (cSCC), cSCC on the lips, annual percent changes (APC), incidence, incidence-based mortality

## Abstract

**Objective:**

This study provided a systematic analysis of the trend in incidence and incidence-based mortality for cutaneous squamous cell carcinoma (cSCC) on the lips in the USA using demographic characteristics from the Surveillance, Epidemiology, and End Results (SEER) database.

**Methods:**

Patients diagnosed with cSCC on the lips between 2000 and 2019 from the 17 registries of the USA were identified. Incidence and incidence-based mortality rates were analyzed using SEER*Stat 8.4.0.1 software. This paper calculated incidence rates and incidence-based mortality rates by 100,000 person-years for sex, age, race, SEER registries, median household income ($/year), rural-urban distribution, and primary site. The annual percent changes (APC) in incidence and incidence-based mortality rates were then calculated using joinpoint regression software.

**Results:**

Among 8,625 patients diagnosed with cSCC on the lips from 2000 to 2019, men (74.67%), white (95.21%), and 60–79 years old were the most common population, and 3,869 deaths from cSCC on the lips occurred. The overall incidence of cSCC on the lips was 0.516 per 100,000 person-years. cSCC on the lip incidence rates were highest among men, white, and patients aged 60–79 years old. cSCC on the lip incidence rates decreased by 3.210%/year over the study period. The incidence of cSCC on the lips has been decreasing in all sexes, ages, high- or low-income households, and urban or rural patients. The overall incidence-based mortality rate of cSCC on the lips during 2000–2019 was 0.235 per 100,000 person-years. cSCC on the lip incidence-based mortality rates were highest among men, whites, and people older than 80 years old. cSCC on the lip incidence-based mortality increased by 4.975%/year over the study period. cSCC on the lip incidence-based mortality rates increased for all sexes, races, ages, primary sites, high- or low-income households, and urban or rural patients during the study period.

**Conclusion:**

Among patients in the USA diagnosed with cSCC on the lips from 2000 to 2019, the overall incidence decreased by 3.210% annually, and incidence-based mortality increased by 4.975%/year. These findings update and supplement the epidemiological information of cSCC on the lips in the USA.

## 1 Introduction

Cutaneous squamous cell carcinoma (cSCC) is a malignant proliferation of the skin epithelium that accounts for 20% to 50% of skin cancers and is the second most common nonmelanoma skin cancer (NMSC) or keratinocytic carcinoma (1–6). cSCC occurs mainly in the head and neck, and more than 50% of newly diagnosed lesions occur in this area (7).

During 2002–2007, the age-standardized incidence rate of cSCC in Europe was nine to 96 cases per 100,000 men and five to 68 cases per 100,000 women (8). According to statistics from 2002, the incidence rate of cSCC in Australia in 2011 was about 499 cases per 100,000 men and 291 cases per 100,000 women, and the mortality rate was two cases per 100,000 people (5). The incidence rate of CSCC in Spain is 40 cases per 100,000 person-years (8). Whereas, the US National Tumor Registry did not include cSCC, and exact US incidence and mortality rates cannot be derived.

The risk factors for cSCC include exposure to the sun, age, fair skin, and immunosuppression (3, 5, 9–11). cSCC is more common in white people and men, with a ratio of 3:1. The incidence rate increased with age, with the median age of onset over 60 years old. Despite a lower prevalence among Hispanic, black, and Asian patients, cSCC is the most common form of skin cancer in these populations. The mortality of cSCC in black patients can be as high as 18% due to delayed diagnosis and poor prognosis (2–5). Most cSCC can be successfully resolved by surgical operation, while some cSCC are at high risk for recurrence, metastasis, and death (12, 13). cSCC on the lips is considered a high-risk skin cancer with a metastasis rate of 11% compared to 1% for other sites. cSCC become a public health problem due to the adverse outcomes it causes.

Therefore, this study provided a systematic analysis of the trend in incidence and the incidence-based mortality for cSCC on the lips in the USA using demographic characteristics from the Surveillance, Epidemiology, and End Results (SEER) database.

## 2 Method

### 2.1 Data sources

This research used the SEER*Stat 8.4.0.1 software to obtain data on cSCC on the lip cases diagnosed during 2000–2019 from SEER-17 registries (“Incidence - SEER Research Data, 17 Registries, November 2021 Sub (2000–2019)”). The SEER-17 Incidence-Based Mortality database provides a convenient and intuitive mechanism for analyzing cSCC on the lip mortality from death certificates (14). In contrast to general mortality rates, incidence-based mortality allows for the classification of deaths based on variables related to cancer occurrence.

### 2.2 Analysis population

This research included patients who were diagnosed with cSCC on the lips between the years 2000 and 2019. Cases were determined by selecting squamous cell tumors (codes 8050-8089) and primary sites on the lips (codes C00.0-00.3, 00.6, 44.0). Demographic characteristics analyzed in this study includes the following variables: sex, race, age at diagnosis (0–19, 20–39, 40–59, 60–79, and 80 years or older) or age at death in the case of incidence-based mortality calculation, SEER registries, median household income ($/year), rural-urban distribution, and primary site.

### 2.3 Statistical analysis

The SEER*stat version 8.4.0.1 software was used to calculate the incidence and incidence-based mortality rates of cSCC on the lips. All rates were adjusted to the 2000 US standard population and expressed by 100,000 person-years. We then used the National Cancer Institute’s Joinpoint Regression program, version 4.9.1.0, to calculate the annual percentage changes (APCs) and 95% confidence intervals (CIs) (15). The Joinpoint Regression software analyzed rates over time and detected significant changes in APCs, then selected the best model with the minimum number of joinpoints and calculated *p*-values using *t*-tests. All statistical tests were two-sided, and *p*-values less than 0.05 were considered statistically significant.

## 3 Results

### 3.1 Baseline characteristics

Between the years 2000 and 2019, 8,625 cases diagnosed as cSCC on the lips in the states recorded by SEER-17 were included in the incidence analysis (**Table 1**). Most patients were men (74.67%), white (95.21%), 60–79 years old (47.14%), and with an external lower lip (80.60%). Incidence-based mortality analysis revealed that, from 2000 to 2019, 3,869 patients with cSCC on the lips died, of whom 2,870 (74.18%) cases were men, 3,773 (97.52%) were white patients, 3,588 (92.8%) were over 60 years old, and 3,115 (80.51%) were external lower lips.

**Table 1:** cSCC on the lip incidence and incidence-based mortality (2000–2019): The SEER-17 registry database.

Characteristic	Incidence				Incidence-based mortality			
	No. of cases	%	Rate	95% CI	No. of deaths	%	Rate	95% CI
Overall	8,625	1.000	0.516	0.505. to 0.527	3,869	1.000	0.235	0.227 to 0.242
Sex								
Male	6,440	0.747	0.867	0.845 to 0.888	2,870	0.742	0.442	0.426 to 0.459
Female	2,185	0.253	0.234	0.224 to 0.244	999	0.258	0.098	0.092 to 0.104
Race	8,625	1.000						
White	8,212	0.952	0.611	0.598 to 0.625	3,773	0.975	0.276	0.268 to 0.285
Black	71	0.008	0.437	0.034 to 0.055	37	0.010	0.027	0.018 to 0.037
American Indian/Alaska Native	30	0.003	0.193	0.126 to 0.279	15	0.004	0.121	0.066 to 0.199
Asian or Pacific Islander	88	0.010	0.055	0.044 to 0.068	34	0.009	0.022	0.015 to 0.031
Unknown	224	0.026			10	0.003		3.851 to 4.186
Age at diagnosis					Age at death			
0–19 years	4	0.000	0.001	0 to 0.002	1	0.000	0.000	0 to 0.001
20–39 years	270	0.031	0.063	0.056 to 0.071	21	0.005	0.005	0.003 to 0.008
40–59 years	2,305	0.267	0.498	0.477 to 0.519	259	0.067	0.054	0.047 to 0.061
60–79 years	4,066	0.471	1.856	1.799 to 1.914	1,345	0.348	0.644	0.61 to 0.68
80+ years	1,980	0.230	3.621	3.463 to 3.785	2243	0.580	4.016	3.851 to 4.186
SEER Registry								
San Francisco-Oakland SMSA	429	0.050	0.453	0.411 to 0.499	189	0.049	0.090	0.164 to 0.22
Connecticut	186	0.022	0.217	0.186 to 0.251	88	0.023	0.094	0.076 to 0.117
Hawaii	82	0.010	0.303	0.244 to 0.374	28	0.007	0.832	0.055 to 0.122
Iowa	861	0.100	1.183	1.104 to 1.266	409	0.106	0.494	0.446 to 0.545
New Mexico	371	0.043	0.845	0.76 to 0.937	183	0.047	0.438	0.377 to 0.507
Seattle (Puget Sound)	687	0.080	0.717	0.664 to 0.773	253	0.065	0.273	0.241 to 0.31
Utah	317	0.037	0.747	0.667 to 0.835	115	0.030	0.290	0.239 to 0.348
Atlanta (Metropolitan)	101	0.012	0.188	0.152 to 0.23	52	0.013	0.112	0.083 to 0.146
Alaska Natives	1	0.000	0.051	0.001 to 0.307	0	0.000	0.000	0 to 0.219
San Jose-Monterey	189	0.022	0.392	0.337 to 0.452	96	0.025	0.204	0.165 to 0.249
Los Angeles	609	0.071	0.326	0.3 to 0.353	264	0.068	0.144	0.127 to 0.163
Rural Georgia	9	0.001	0.288	0.131 to 0.564	3	0.001	0.106	0.022 to 0.322
California excluding SF/SJM/LA	2574	0.298	0.638	0.614 to 0.663	1,070	0.277	0.270	0.254 to 0.286
Kentucky	752	0.087	0.800	0.743 to 0.86	384	0.099	0.424	0.382 to 0.469
Louisiana	522	0.061	0.542	0.496 to 0.591	258	0.067	0.285	0.251 to 0.322
New Jersey	479	0.056	0.239	0.218 to 0.262	236	0.061	0.115	0.101 to 0.131
Greater Georgia	446	0.052	0.361	0.328 to 0.397	241	0.062	0.212	0.186 to 0.241
Median household income								
<$75,000	6,383	0.740	0.562	0.548 to 0.576	2,943	0.761	0.265	0.255 to 0.275
$75,000+	2,238	0.259	0.417	0.4 to 0.435	926	0.239	0.171	0.16 to 0.182
Unknown	4	0.000						
Urban and rural								
Urban	6747	0.782	0.460	0.449 to 0.471	2,940	0.760	0.204	0.197 to 0.212
Rural	1,873	0.217	0.912	0.87 to 0.955	929	0.240	0.445	0.416 to 0.474
Unknown	5	0.001	0.258	0.084 to 0.615	0	0.000		0 to 0.219
Primary site								
External upper lip	1,022	0.118	0.061	0.058 to 0.065	444	0.115	0.027	0.025 to 0.03
External lower lip	6,952	0.806	0.415	0.406 to 0.425	3,115	0.805	0.189	0.182 to 0.195
External lip, not otherwise specified	314	0.036	0.019	0.017 to 0.021	131	0.034	0.008	0.007 to 0.010
Commissure of lip	337	0.039	0.02	0.018 to 0.022	179	0.046	0.011	0.009 to 0.013

cSCC, cutaneous squamous cell carcinoma; SEER, Surveillance, Epidemiology, and End Results.

No. (%): The number of deaths was based on cases diagnosed during 2000–2019.

### 3.2 Incidence rates and trends over time

The overall incidence of cSCC on the lips during 2000–2019 was 0.516 (95% CI, 0.505 to 0.527) per 100,000 person-years. cSCC on the lip incidence rates were highest among men (0.867 (95% CI, 0.845 to 0.888)), white (0.437 (95% CI, 0.034 to 0.055)), patients 60–79 years old (1.856 (95% CI, 1.799 to 1.914)), and external lower lip patients (0.415 (95% CI, 0.406 to 0.425)). When compared to states in the SEER-17 registries, the incidence was the highest in Iowa (1.183 (95% CI, 1.104 to 1.266)) and the lowest in Alaska Natives (0.051 (95% CI, 0.001 to 0.307)) (**Table 1**).

**Figure 1:**
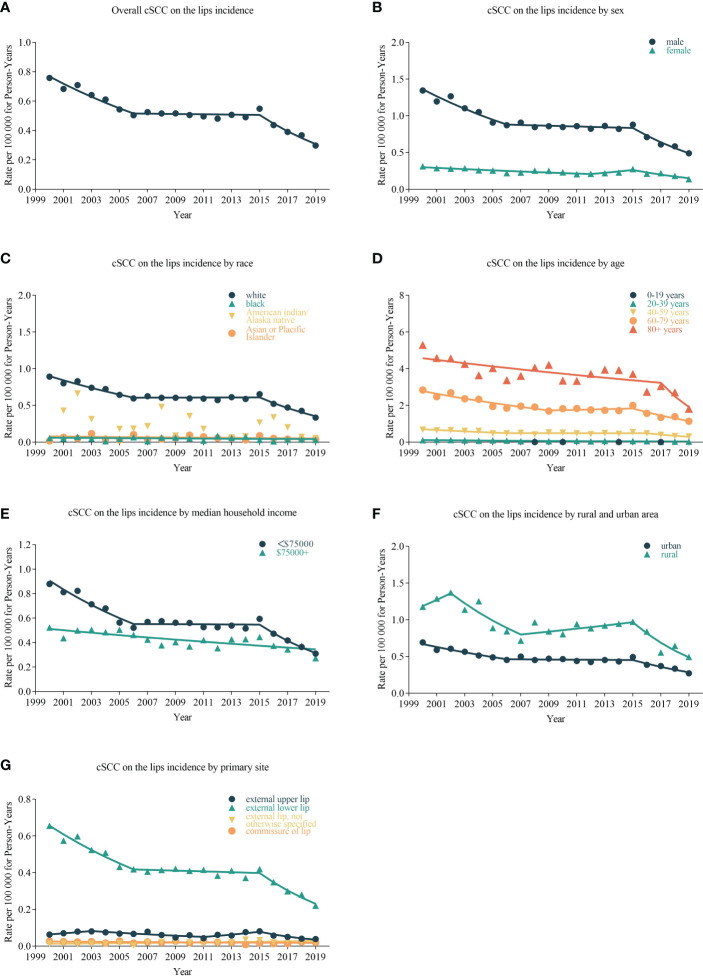
Trends in annual cSCC on the lip incidence rates. Data markers delegate observed incidence rates (per 100,000 person-years). The slope of the line indicates the annual percentage change (APC). **(A)** The overall incidence and trend of cSCC on the lips patients; the line graphs are classified into different groups, including sex **(B)**, race **(C)**, age **(D)**, median household income **(E)**, urban and rural area **(F)**, and primary site **(G)**.

cSCC on the lip incidence rates decreased by −3.210% (95% CI, −3.994 to −2.420; *p* < 0.001) per year over the study period, with an incidence rate of 0.022 in 2000 and 0.294 in 2019 per 100,000 person-years (**Table 2**). Rates did not decrease significantly during 2005–2009 (APC = −0.774%, (95% CI, −3.539 to 2.07; *p* = 0.446)) and 2010–2014 (APC = −3.396% (95% CI, −2.766 to 2.032; *p* = 0.636)) but decreased by −13.153% (95% CI, −17.558 to −8.513; *p* = 0.003) per year during 2015–2019. The incidence of cSCC on the lips has been decreasing in all sex and ages (**Figure 1**). From 2000 to 2019, there was a significant downward trend in the incidence rates for high- or low-income households, urban or rural patients, and external lower lip patients (APC = −3.766% (95% CI, −4.596 to −2.929; *p* < 0.001)). Among low-income households, the incidence rate decreased significantly from 2000 to 2004 (APC = −6.210% (95% CI, −9.715 to −2.568; *p* = 0.013]) and among high-income households, from 2005 to 2009 (APC = −6.362% (95% CI, −11.184 to −1.278; *p* = 0.029)). In urban areas, there was a significant decrease in prevalence in 2000–2004 (APC = −6.245% (95% CI, −10.608 to −1.668; *p* = 0.023)) and 2015–2019 (APC = −12.574% (95% CI, −17.555 to −7.293; *p* = 0.005)); in rural areas in 2015–2019 (APC = −15.288% (95% CI, −25.641 to −3.494; *p* = 0.027)), the incidence rate also decreased significantly. The incidence has been decreasing in most subgroups of SEER registries except for Seattle (Puget Sound) and Utah. However, it has not changed recently among blacks and Asians or Pacific Islanders and decreased significantly among whites (APC = −3.136% (95% CI, −3.978 to −2.287; *p* = 0.030)).

**Table 2:** Trends in cSCC on the lip incidence rates (2000–2019): The SEER-17 registry database.

Characteristic	Overall			2000–2004			2005–2009			2010–2014			2015–2019		
	APC	95% CI	*p-*value	APC	95% CI	*p-*value	APC	95% CI	*p-*value	APC	95% CI	*p-*value	APC	95% CI	*p-*value
Overall	−3.21	−3.994 to −2.420	<0.001	−4.831	−8.299 to −1.232	0.0241	−0.774	−3.539 to 2.07	0.446	−3.396	−2.766 to 2.032	0.636	−13.153	−17.558 to −8.513	0.003
Sex															
Male	−3.702	−4.507 to −2.889	<0.001	−5.575	−10.189 to −0.724	0.036	−1.412	−3.929 to 1.171	0.179	−0.579	−2.997 to 1.899	0.507	−12.998	−17.316 to −8.454	0.003
Female	−2.211	−3.038 to −1.276	<0.001	−3.887	−7.138 to −0.523	0.035	1.047	−6.441 to 9.135	0.696	0.447	−5.111 to 6.33	0.819	−13.941	−21.493 to −5.663	0.014
Race															
White	−3.136	−3.978 to −2.287	0.03	−4.894	−8.477 to −1.171	0.025	−1.134	−4.039 to 1.859	0.311	0.066	−2.673 to 2.883	0.944	−14.255	−18.277 to −10.034	0.002
Black	−1.989	−5.891 to 2.074	0.313	−15.855	−52.279 to 48.372	0.404	4.75	−35.977 to 71.382	0.784	10.024	−24.837 to 61.054	0.483	7.093	−29.445 to 62.552	0.637
American Indian/Alaska Native							4.687	−48.049 to	0.849				−36.611	−59.32 to	0.047
Asian or Pacific Islander	−2.377	−5.950 to 1.332	0.192	27.046	−43.022 to 183.282	0.412	−7.404	−48.586 to 66.763	0.705	−20.568	−31.283 to −8.183	0.015	−19.15	−34.721 to 0.135	0.051
Age at diagnosis															
0–19 years															
20–39 years	−6.607	−8.171 to −5.017	<0.001	−9.144	−22.857 to 7.007	0.159	−14.068	−26.85 to 0.947	0.058	−3.491	−20.528 to 17.199	0.601	−20.678	−39.933 to 4.75	0.077
40–59 years	−2.722	−3.696 to −1.739	<0.001	−2.342	−4.381 to −0.259	0.037	0.982	−8.447 to 11.382	0.772	−2.451	−8.807 to 4.348	0.326	−14.541	−17.008 to −12.001	0
60–79 years	−3.433	−4.214 to −2.646	<0.001	−4.351	−9.621 to 1.225	0.088	−2.359	−7.44 to 3.001	0.25	−1.806	−3.056 to −0.539	0.02	−12.039	−18.854 to −4.651	0.015
80+ years	−2.734	−3.859 to −1.597	<0.001	−7.858	−12.002 to −3.518	0.011	2.717	−6.251 to 12.542	0.419	4.962	1.316 to 8.738	0.022	−12.84	−24.66 to 0.835	0.058
SEER Registry															
San Francisco-Oakland SMSA	−4.534	−6.600 to −2.422	<0.001	−3.752	−20.726 to 16.857	0.575	−0.303	−20.958 to 25.75	0.969	−1.554	−27.531 to 33.735	0.881	−12.556	−35.073 to 17.771	0.247
Connecticut	−0.331	−3.646 to 3.098	0.839	−7.847	−33.118 to 26.972	0.477	−9.984	−32.913 to 20.781	0.338	4.91	−36.754 to 74.022	0.783	−17.99	−47.702 to 28.603	0.255
Hawaii				29.229	−18.976 to 106.113	0.179				−5.946	−36.311 to 38.897	0.651	−15.5421	−46.892 to 34.314	0.33
Iowa	−2.987	−4.265 to −1.691	<0.001	3.503	−8.972 to 17.689	0.456	−5.146	−22.59 to 16.229	0.469	−1.837	−16.227 to 15.026	0.735	−8.998	−19.026 to 2.271	0.082
New Mexico	−10.502	−12.399 to −8.563	<0.001	−2.985	−18.996 to 16.19	0.63	−10.776	−34.535 to 21.605	0.326	−14.693	−36.905 to 15.338	0.192	8.183	−16.948 to 40.919	0.414
Seattle (Puget Sound)	3.071	0.895 to 5.294	0.008	−2.715	−17.354 to 14.518	0.628	4.62	−7.012 to 17.708	0.31	8.735	−21.035 to 49.727	0.466	−5.674	−32.873 to 32.546	0.623
Utah	0.958	−1.935 to 3.936	0.5	11.508	−1.501 to 26.236	0.068	8.416	−19.243 to 45.548	0.447	23.7591	1.691 to 50.616	0.041	−13.629	−38.641 to 21.579	0.266
Atlanta (Metropolitan)	−3.104	−7.521 to 1.523	0.173	28.913	−39.408 to 174.271	0.363	−10.308	−44.924 to 46.063	0.529	−15.934	−51.981 to 47.175	0.397	5.727	−40.114 to 86.66	0.776
Alaska Natives															
San Jose-Monterey	−4.32	−7.129 to −1.426	0.006	−2.906	−19.725 to 17.437	0.656	−12.485	−25.438 to 2.719	0.077	2.674	−12.997 to 21.168	0.647	−21.707	−54.401 to 34.426	0.245
Los Angeles	−3.628	−5.407 to −1.814	0.001	−17.937	−28.785 to −5.437	0.021	6.34	−13.709 to 31.048	0.418	−3.426	−13.645 to 8.001	0.394	−12.959	−24.283 to 0.058	0.051
Rural Georgia															
California excluding SF/SJM/LA	−3.783	−4.741 to −2.816	<0.001	−9.412	−12.452 to −6.267	0.003	−1.711	−11.589 to 9.27	0.64	−3.734	−5.874 to −1.545	0.013	−14.0081	−25.976 to −0.104	0.049
Kentucky	−4.019	−5.915 to −2.085	<0.001	−0.794	−22.295 to 26.655	0.924	−2.3221	−20.242 to 19.624	0.737	5.247	0.558 to 10.154	0.038	−25.477	−34.86 to −14.743	0.006
Louisiana	−2.496	−4.330 to −0.606	0.012	−2.181	−24.285 to 26.376	0.802	6.858	3.688 to 10.126	0.006	4.759	−17.499 to 33.02	0.579	−9.946	−27.589 to 11.995	0.224
New Jersey	−3.037	−4.902 to −1.135	0.004	−5.267	−14.311 to 4.731	0.185	17.253	1.584 to 35.34	0.039	−9.085	−23.522 to 8.079	0.178	−16.147	−36.424 to 10.598	0.136
Greater Georgia	−4.681	−6.883 to −2.427	<0.001	−6.353	−20.419 to 10.199	0.289	−4.159	−17.633 to 11.519	0.438	0.882	−5.575 to 7.78	0.701	−17.259	−43.975 to 22.197	0.22
Median household income															
<$75,000	−3.687	−4.604 to −2.761	<0.001	−6.21	−9.715 to −2.568	0.013	0.9321	−3.124 to 5.159	0.524	−1.43	−4.057 to 1.269	0.188	−14.669	−17.432 to −11.813	0.001
$75,000+	−2.058	−2.841 to −1.270	<0.001	−0.249	−7.65 to 7.745	0.925	−6.362	−11.184 to −1.278	0.029	3.226	−4.889 to 12.033	0.305	−9.014	−18.057 to 1.027	0.064
Urban and rural															
Urban	−3.036	−3.802 to −2.264	<0.001	−6.245	−10.608 to −1.668	0.023	−0.833	−5.496 to 4.06	0.619	−1.118	−4.537 to 2.424	0.384	−12.574	−17.555 to −7.293	0.005
Rural	−3.288	−4.576 to −1.982	<0.001	−0.054	−8.434 to 9.093	0.986	0.404	−11.173 to 13.49	0.923	2.974	−2.462 to 8.713	0.184	−15.288	−25.641 to −3.494	0.027
Primary site															
External upper lip	−1.978	−3.57 to −0.359	0.02	4.876	−2.934 to 13.314	0.145	−7.708	−22.354 to 9.7	0.236	8.282	−8.711 to 28.44	0.235	−17.564	−24.682 to −9.773	0.006
External lower lip	−3.766	−4.596 to −2.929	<0.001	−5.842	−10.057 to −1.43	0.025	−0.552	−2.875 to 1.827	0.51	−2.046	−6.247 to 2.342	0.23	−13.958	−17.682 to −10.066	0.002
External lip, not otherwise specified	4.284	1.577 to 7.062	0.004	−4.911	−19.528 to 12.36	0.408	13.295	−23.775 to 68.394	0.39	21.223	−9.946 to 63.179	0.131	−8.28	−25.769 to 13.329	0.284
Commissure of lip	−1.981	−3.835 to −0.091	0.041	−8.53	−20.135 to 4.762	0.128	0.852	−19.418 to 26.223	0.912	−13.3	−41.105 to 27.63	0.325	10.524	−9.205 to 34.54	0.204

cSCC, cutaneous squamous cell carcinoma; APC, annual percent change; SEER, Surveillance, Epidemiology, and End Results.

### 3.3 Incidence-based mortality rates and trends over time

The overall incidence-based mortality rate of cSCC on the lips during 2000–2019 was 0.235 (95% CI, 0.227 to 0.242) per 100,000 person-years. cSCC on the lip incidence-based mortality rates were highest among men (0.442 (95% CI, 0.426 to 0.459%)), whites (0.276 (95% CI, 0.268 to 0.285)), people older than 80 years old (4.016 (95% CI, 3.851 to 4.186)), and external lower lip patients (0.189 (95% CI, 0.182 to 0.195)). When compared to states in the SEER-17 registries, incidence-based mortality was highest in Hawaii (0.832 (95% CI, 0.055 to 0.122)) and lowest in Alaska Natives (0 (95% CI, 0 to 0.219)).

**Figure 2:**
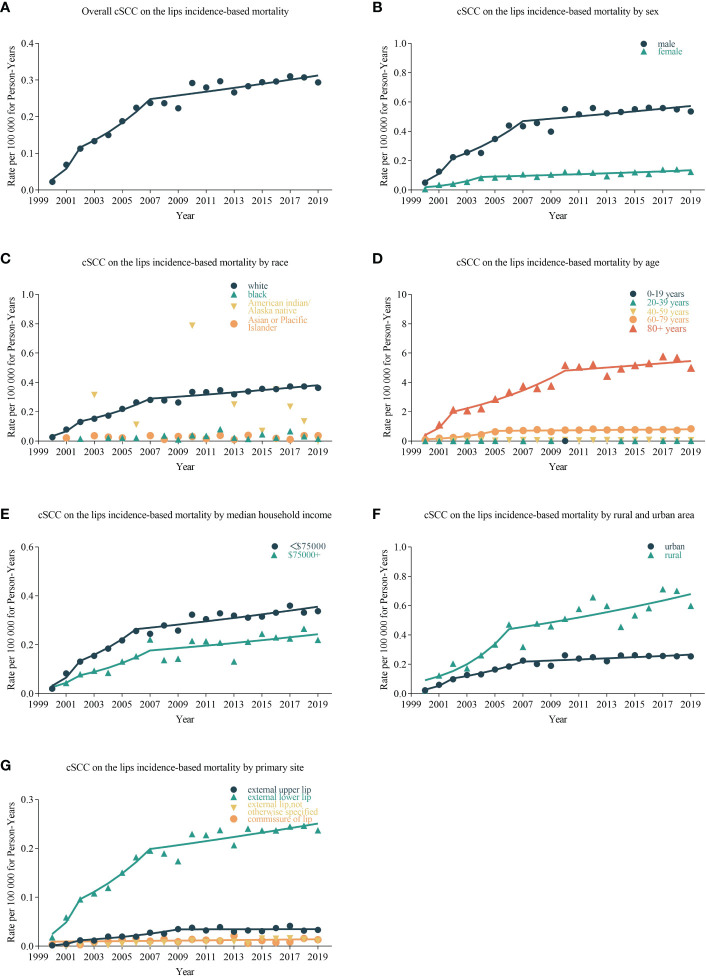
Trends in annual cSCC on the lip incidence-based mortality rates. Data markers represent observed incidence-based mortality rates (per 100,000 person-years). The slope of the line shows the annual percentage change (APC). **(A)** The overall incidence-based mortality and trend of cSCC on the lips patients; line graphs are divided into different groups, including sex **(B)**, race **(C)**, age **(D)**, median household income **(E)**, urban and rural area **(F)**, and primary site **(G)**.

cSCC on the lip incidence-based mortality increased by 4.975% (95% CI, 2.942 to 7.048; *p* < 0.001) per year over the study period (**Table 3**). cSCC on the lip incidence-based mortality rates increased for all sexes, races, and ages during the study period (**Figure 2**). High-income (APC = 4.782 [95% CI, 2.810–6.792, *p* < 0.001)), low-income (APC = 5.856 (95% CI, 3.471–8.296; *p* < 0.001)), urban (APC = 4.732 (95% CI, 2.663 to 6.843; *p* < 0.001)), rural (APC = 6.331 (95% CI, 3.877 to 8.842; *p* < 0.001]) patients, external upper lip (APC = 5.154 (95% CI, 2.362 to 8.022; *p* = 0.001)), and external lower lip (APC = 4.909 (95% CI, 2.911 to 6.945; *p* < 0.001)) showed a significant increase in mortality. In urban areas, there was a significant increase in morbidity-based mortality in 2000–2004 (APC = 37.693 (95% CI, 0.675 to 88.322; *p* = 0.047)) and a significant decrease in morbidity-based mortality in 2015–2019 (APC = −0.732 (95% CI, −1.244 to −0.218; *p* = 0.02)).

**Table 3:** Trends in cSCC on the lip incidence-based mortality rates (2000–2019): The SEER-17 registry database.

Characteristic	Overall			2000–2004			2005–2009			2005–2010			2015–2019		
	APC	95% CI	*p-*value	APC	95% CI	*p-*value	APC	95% CI	*p*-value	APC	95% CI	*p*-value	APC	95% CI	*p*-value
Overall	4.975	2.942 to 7.048	<0.001	37.062	−1.225 to 90.189	0.055	3.653	−4.76 to 12.809	0.27	−1.08	−5.355 to 3.388	0.491	0.307	−2.683 to 3.389	0.768
Sex															
Male	4.747	2.655 to 6.881	<0.001	32.778	−7.681 to 90.968	0.089	2.636	−8.451 to 15.065	0.521	−0.601	−4.302 to 3.244	0.648	−0.781	−2.321 to 0.784	0.209
Female	4.583	2.533 to 6.674	<0.001	43.3554	2.149 to 101.181	0.043	4.442	−4.809 to 14.591	0.233	−4.641	−12.563 to 3.998	0.18	2.896	−7.927 to 14.991	0.474
Race															
White	5.354	3.338−7.409	<0.001	37.273	−0.526 to 89.435	0.052	3.793	−4.891 to 13.27	0.268	−0.219	−3.726 to 3.416	0.858	0.845	−1.5 to 3.246	0.338
Black										−13.313	−64.081 to 109.213	0.641	−15.131	−55.489 to 61.821	0.478
American Indian/Alaska Native															
Asian or Pacific Islander										6.735	−37.856 to 83.322	0.727			
Age at death															
0–19 years															
20–39 years				36.935	−6.163 to 99.826	0.077									
40–59 years	3.973	1.600−6.400	0.002				2.463	−31.829 to 54.004	0.861	−1.837	−19.591 to 19.837	0.787	−7.117	−23.364 to 12.574	0.309
60–79 years	5.831	3.747−7.957	<0.001	39.206	19.474 to 62.197	0.006	0.85	−9.589 to 12.494	0.821	0.679	−3.215 to 4.731	0.623	1.646	−3.21 to 6.746	0.366
80+ years				30.282	−18.736 to 108.867	0.172	6.029	−0.938 to 13.485	0.071	−2.133	−8.108 to 4.23	0.356	0.039	−7.09 to 7.715	0.988
SEER Registry															
San Francisco-Oakland SMSA							15.686	−20.184 to 67.678	0.3	−0.299	−11.012 to 11.704	0.938	−4.193	−23.728 to 20.345	0.592
Connecticut	3.877	−0.591 to 8.546	0.086	32.334	−16.074 to 108.664	0.145	3.366	−38.208 to 72.912	0.851	3.635	−32.603 to 59.357	0.809	−7.873	−42.747 to 48.244	0.621
Hawaii													28.389	−21.558 to 110.139	0.205
Iowa							−8.667	−27.319 to 14.772	0.296	−4.926	−24.293 to 19.394	0.531	7.784	2.217 to 13.654	0.021
New Mexico							1.548	−29.734 to 46.756	0.903	8.382	−14.038 to 36.651	0.35	−3.124	−29.914 to 33.907	0.775
Seattle (Puget Sound)	7.31	4.503 to 10.193	<0.001	28.306	3.237 to 59.462	0.036	−4.372	−34.864 to 40.394	0.736	0.101	−23.265 to 30.581	0.991	1.227	−24.365 to 35.48	0.902
Utah							22.054	−6.292 to 58.976	0.096	−11.9421	−38.328 to 25.733	0.338	−14.501	−33.792 to 10.412	0.146
Atlanta (Metropolitan)							−6.336	−48.534 to 70.463	0.751	18.837	−47.955 to 171.347	0.553	15.155	−33.678 to 99.944	0.475
Alaska Natives															
San Jose-Monterey							12.564	−39.973 to 111.085	0.591	−4.051	−34.154 to 39.815	0.75	−13	−27.878 to 4.946	0.099
Los Angeles							−4.354	−31.927 to 34.387	0.705	−8.697	−30.333 to 19.657	0.363	6.942	−17.524 to 38.665	0.471
Rural Georgia															
California excluding SF/SJM/LA	4.806	2.448 to 7.217	<0.001	34.859	−7.02 to 95.6	0.083	5.635	−11.923 to 26.693	0.408	0.54	−9.26 to 11.398	0.878	−1.725	−10.647 to 8.088	0.602
Kentucky	5.521	2.805 to 8.308	<0.001	25.151	−33.451 to 135.359	0.34	7.139	−19.293 to 42.227	0.495	2.2321	−20.1 to 30.806	0.794	−0.044	−25.169 to 33.517	0.996
Louisiana	5.114	2.191 to 8.121	0.002	20.139	−13.498 to 66.856	0.174	14.947	−12.105 to 50.325	0.197	−4.862	−27.362 to 24.607	0.598	0.21	−20.726 to 26.674	0.979
New Jersey							3.237	−26.664 to 45.329	0.786	−0.882	−14.9 to 15.444	0.865	−0.911	−13.159 to 13.065	0.84
Greater Georgia							4.13	−19.372 to 34.482	0.649	−2.426	−9.948 to 5.724	0.402	0.044	−14.064 to 16.467	0.993
Median household income															
<$75,000	4.782	2.810−6.792	<0.001	38.913	0.059 to −2.409	97.733	4.022	0.187 to −3.364	11.972	−0.354	0.763 to −3.696	3.104	1.381	0.451 to −3.61	6.63
$75,000+	5.856	3.471−8.296	<0.001	29.054	0.076 to −4.806	74.956	0.588	0.952 to −24.475	33.967	−3.653	0.598 to −21.241	17.862	−0.528	0.869 to −9.466	9.294
Urban and rural															
Urban	4.732	2.663 to 6.843	<0.001	37.693	0.047 to 0.675	88.322	3.331	0.458 to −8.614	16.836	−0.681	0.8 to −8.183	7.434	−0.732	0.02 to −1.244	−0.218
Rural	6.331	3.877 to 8.842	<0.001	33.233	0.137 to −15.274	109.512	5.974	0.414 to −12.832	28.836	−1.496	0.794 to −16.738	16.535	4.07	0.402 to −8.647	18.556
Primary site															
External upper lip	5.154	2.362 to 8.022	0.001	58.053	7.165 to 133.108	0.033	15.667	3.339 to 29.466	0.026	−3.998	−14.427 to 7.703	0.341	−0.207	−14.247 to 16.132	0.968
External lower lip	4.909	2.911 to 6.945	<0.001	34.29	−5.584 to 91.002	0.076	2.893	−7.318 to 14.228	0.449	0.155	−6.453 to 7.229	0.947	0.412	−1.785 to	0.596
External lip, not otherwise specified							4.817	−20.721 to 38.582	0.629	−12.549	−28.42 to 6.843	0.123	−8.561	−18.489 to 2.577	0.089
Commissure of lip	2.272	−1.407 to 6.088	0.214	37.867	3.774 to 83.162	0.037	−7.871	−26.943 to 16.18	0.343	0.555	−41.162 to 71.848	0.976	10.676	−15.94 to 45.719	0.325

cSCC, cutaneous squamous cell carcinoma; APC, annual percent change; SEER, Surveillance, Epidemiology, and End Results.

## 4 Discussion

The lips are the anatomical junction of two distinct groups of cancers. Lip squamous cell carcinoma (lip SCC) is more aggressive than cSCC but less aggressive than oral mucosal squamous cell carcinoma (16). The external lower lip was the location with the highest risk of metastasis (17, 18). Therefore, this study was conducted to systematically analyze the trends in incidence and incidence-based mortality for cSCC on the lips using the SEER database.

Since the 1980s, researchers have demonstrated that UVR can cause skin damage and increase the risk of skin cancer. In 1992, the International Agency for Research on Cancer classified solar radiation as the first category of carcinogenic hazard, which is known to cause human cancer. In 2009, the World Health Organization classified UVR tanning devices as a Group I carcinogen based on evidence related to indoor tanning and an increased risk of skin cancer (19).

This study mainly found that the age-adjusted incidence rate of cSCC on the lips decreased significantly from 2000 to 2019, while the incidence-based mortality rate increased significantly. People with fair skin have less melanin and less protection against UVR (20–22). Consistent with the results of previous studies, cSCC on the lips was most common in white people (22) and less common in black people, Indians, and Asians (23, 24). It is more common in men than in women, with a male-to-female ratio of up to 4:1 (22, 25). The high incidence in men may be related to the fact that men were exposed to the sun for a relatively long time for working (26). Women have more knowledge of skincare and sun protection, use sunscreen lotion, and seek shade more frequently than men (27). The decrease in incidence in men may be due to the strengthening of sun protection education, the increase in the proportion of wearing sun protection clothing, and the increase in the number of people using sunscreen (28, 29).

The reduction of the incidence rate may also be influenced by other factors. Actinic keratosis (AK) is considered a precancerous lesion caused by prolonged exposure of the skin to UVR and is one of the most common causes of dermatologic treatment, which can progress to invasive cSCC (30, 31). Advanced age, fair skin, and prolonged sun exposure are major risk factors for AK (32, 33). The incidence of AK was increasing due to extended life expectancy and inappropriate sun exposure behaviors. Although the exact prevalence of AK is unknown, it affects 1%–44% of the adult population worldwide (32). There were more than 3,500 new cases of lip cancer in the USA, 90% of which were cSCC. A recent study showed that the prevalence of AC was 31.3% among people over 45 years old in northwestern Spain (34). The treatments for AK include cryotherapy, topical treatments with or without photodynamic therapy (PDT), and surgery and laser therapy which are less used (32). The actual definition of AK remains controversial; it is also defined as carcinoma *in situ* by some experts. AC (35) is a precursor of cSCC on the lips (36). Similar to AK, AC is a precancerous lesion caused by prolonged sun exposure or UVR that is most commonly on the lower lip along the vermilion border. Other risk factors include increasing age, especially over 60 years, working outdoors for more than 25 years, or a history of NMSC (37). The prevalence of AC is higher in people with fair skin and in areas with high UV exposure near the equator. Men have a higher incidence of AC than women, probably because they are exposed to relatively more sunlight and use less lip balm and cosmetics (38).

The improvement of awareness of precancerous lesions, early diagnosis abilities, and more timely treatment methods can cure precancerous lesions at an early stage and prevent precancerous lesions from developing into cSCC. The reduced incidence may be associated with this. Immunosuppression is a high-risk factor for cSCC, which may lead to the development of invasive cancer and the metastasis of cSCC (10–13, 39–42). Patients with kidney or heart transplantation are 65 times more likely to develop cSCC than general populations (41). In recent years, the use of immunomodulators in the treatment of autoimmune diseases, allergic diseases, and cancer has become popular (43–46). This may also be one of the factors leading to the increase in mortality. The increase in mortality may also be related to the aging of the population and the prolongation of the lifespan of patients with chronic diseases (47). cSCC on the lips is considered a high-risk skin cancer with a metastasis probability of 11%, compared to 1% for other body sites (36), making it critical to identify and appropriately manage these potentially malignant precursor lesions.

cSCC on the lips is prevalent in people over 40 years of age, with the highest incidence in 60–79 years, and incidence-based morbidity increases over 80 years old. The elderly were relatively vulnerable, and the 5-year disease-specific survival rate of patients aged over 75 years old, especially those over aged 85 years old, is relatively poor (23). A study has found that old age is not a risk factor for poorer survival in metastatic cSCC on the lips, which might be limited to metastatic cSCC on the lips disease (48). The trend analysis in this study reflected the high morbidity of both metastatic and nonmetastatic disease in elderly patients. There were more risk factors in elderly patients that may lead to high incidence and mortality in elderly patients (25). Elderly patients are more likely to suffer from tumors with large tumor diameters, low differentiation, deep tumor invasion, and more lymph node metastasis than young people. The risk of postoperative complications is high in elderly patients, with large postoperative defects and difficult wound healing. The poor mobility and weakness of the elderly make regular follow-up more difficult and the prognosis worse (25, 49).

Incidence and incidence-based mortality rates were higher among low-income and rural dwellers than among high-income and urban dwellers. Skin cancer was also a major economic burden in the USA (19). The cost of treating melanoma and NMSC was estimated at $1.7 billion per year (50, 51). The cost of lost productivity was estimated to be $3.8 billion (52). Prevention and early detection are effective ways to reduce the burden of disease and the cost of treatment (19, 53). A study conducted in Minnesota, USA, found that compared with urban dwellers, rural dwellers spent more time outdoors, used sun lotion less frequently, and had less awareness of sun protection (54). There were higher standards of education and income in urban dwellers than rural dwellers (42). Whites and people with high education levels know more about skin cancer and sunscreen, including using sunscreen, looking for shade, and wearing sunscreen clothes (27, 42, 55).

There are several limitations to this study. Some clinical data, such as tobacco and alcohol intake (39), staging and grading information, tumor depth (56), immunosuppression, HPV status, and family history of skin cancer, were lacking in the SEER database, thus limiting the ability of our results to exactly reflect the national patient population. Therefore, there may be a bias in the clinicopathological characteristics of tumors and specific risk factors for a poorer prognosis. The distribution of cases throughout race, age, and geographic regions is not equally represented. These restrictions should be kept in mind when using data collected through these registries. Nevertheless, the SEER database provides powerful statistical capabilities in evaluating the demographic characteristics of cSCC on the lips. Multicentered effort and additional risk factors are needed to further understand the incidence and incidence-based mortality of cSCC on the lips.

This study analyzed trends in incidence and incidence-based mortality in patients with cSCC on the lips from 2000 to 2019 and update and supplemented the epidemiological information on this type of cutaneous cancer. The incidence and incidence-based mortality in patients with cSCC on the lips were decreasing, but the mortality rate was increasing. The decrease in incidence might be related to the improvement of sun protection awareness with the increase in income and education levels, as well as timely diagnosis and treatment of precancerous lesions, thus preventing further deterioration of cSCC. The decline in men’s incidence was related to the improvement in male sunscreen protection awareness. The increased mortality may be related to the aging of the population and the use of immunosuppressive drugs that lead to the development of cSCC into invasive cancer.

## Data availability statement

The datasets presented in this study can be found in online repositories. The names of the repository/repositories and accession number(s) can be found in the article/supplementary material.

## Author contributions

JZ designed the study. JZ and QY contributed to data analysis. JZ wrote the initial draft of the manuscript. JZ, QY, JW, RY, YL, XZ, XC, BW, and NZ reviewed and edited the manuscript. All authors contributed to the article and approved the submitted version.
